# Genome Instability-Related miRNAs Predict Survival, Immune Landscape, and Immunotherapy Responses in Gastric Cancer

**DOI:** 10.1155/2021/2048833

**Published:** 2021-11-01

**Authors:** Yaqiong Liu, Lin Cheng, Wei Huang, Xin Cheng, Weijun Peng, Dazun Shi

**Affiliations:** ^1^Department of Integrated Traditional Chinese & Western Medicine, The Second Xiangya Hospital, Central South University, Changsha, Hunan 410011, China; ^2^Regenerative Medicine Institute (REMEDI), CURAM, National University of Ireland Galway, H91TK33, Galway, Ireland; ^3^Department of Integrated Traditional Chinese and Western Medicine, Xiangya Hospital, Central South University, Changsha, Hunan 410008, China; ^4^Department of Gynecology and Obstetrics, Xiangya Hospital, Central South University, Changsha, Hunan 410008, China

## Abstract

**Background:**

Increasing evidence suggests that microRNAs (miRNAs) are involved in genome instability (GI) and drive the occurrence of tumors. However, the role of GI-related miRNAs in gastric cancer (GC) remains largely unknown. Herein, we developed a novel GI-related miRNA signature (GIMiSig) and further investigated its role in prognosis, the immune landscape, and immunotherapy responses in GC patients.

**Methods:**

An analysis of somatic mutation data on 434 gastric cancer cases from The Cancer Genome Atlas (TCGA) database was performed, thereby generating genome stability (GS) and GI groups. By detecting differentially expressed miRNAs between the GS and GI groups that were associated with overall survival, 8 miRNAs were identified and used to construct the GIMiSig.

**Results:**

The GIMiSig showed high accuracy in detecting GC patients. Using GIMiSig to stratify the patients into the high- and low-risk subgroups to predict survival outperformed the use of regular clinical features such as age, gender, or disease stage. Patients with low risk had a more favorable survival time than those with high risk. More importantly, the high-risk patients were associated with decreased UBQLN4 expression, higher accumulation of immune cells, lower Titin (TTN) mutation frequency, worse immunotherapy efficacy, and cancer-associated pathways. Conversely, the low-risk patients were characterized by UBQLN4 overexpression, lower fraction of immune cells, higher TTN mutation frequency, better response to immunotherapy, and GI-related pathways.

**Conclusion:**

In summary, we constructed a novel GIMiSig that could stratify GC patients into distinct risk groups that have different survival outcomes and immunotherapy efficacy. The results may provide new clues for improving GC outcomes.

## 1. Introduction

Gastric cancer (GC) is the second most common cause of cancer-related mortality worldwide, causing severe economic and social burdens [1]. Although multiple strategies such as surgery, chemotherapy, and radiotherapy have been used, the outcomes of GC patients remain poor, partly due to the lack of early detection and the high frequency of recurrence [2]. Recently, immune checkpoint inhibitors (ICIs) targeting immune checkpoints, especially programmed cell death-1 (PD-1) and its ligand (PD-L1), have shown encouraging results in several cancer types [3]. However, immunotherapy efficacy in GC remains poor because of the heterogeneity among GC subtypes and the complex interactions within the immune system [4]. Therefore, there is a need to identify novel biomarkers for risk stratification, immune landscape assessment, and prediction of therapeutic effects.

Genomic instability (GI) is regarded as a hallmark of malignant tumors because it results in genomic alteration of cells, thereby facilitating cell proliferation without restraint [5]. Unsurprisingly, GI is also a crucial step in driving tumorigenesis in GC patients [6]. The mechanisms of GI in cancer remain mysterious, but it is widely accepted that miRNAs are involved in the regulation of GI, and they can be used to predict cancer outcomes. For instance, Wang et al. built a risk model involving 10 GI-related miRNAs that predicted prognosis and chemotherapy responses in ovarian cancer [7]. In malignant glioma, miR-100 led to GI by impairing cell responses to DNA damage [8]. In hepatocellular carcinoma, miR-24, miR-103, and miR-107 are considered to be carcinogenic factors because they enhance GI in multiple ways, such as influencing the cell cycle and reducing DNA damage repair [9, 10]. Additionally, in breast cancer, miR-155 overexpression provoked GI by disturbing telomere homeostasis, contributing to an unfavorable prognosis [11]. Although emerging research has shown that miRNAs participate in GI in various tumor types, the roles of GI-related miRNAs in GC remain unclear.

Additionally, the tumor microenvironment (TME) has become a research hotspot in cancer. The TME is a complex system that consists of multiple cells, proteases, and cytokines, and it impacts tumorigenesis and immunotherapy responses. More and more evidence has confirmed the crucial roles of miRNAs in the TME. For example, Chou et al. found that miR-29b modulated the TME to prevent the metastasis of breast cancer [12]. Furthermore, miR-9 modulates the TME by stimulating angiogenesis [13]. However, research on the function of GI-related miRNAs in the TME is very scarce. Thus, we aimed to investigate the interaction between GI-related miRNAs and the TME in GC.

Currently, some biomarkers have been found to correlate with the prognosis and treatment of GC. UBQLN4 contributed to the occurrence of GI and was shown to inhibit GC [14–16]. TTN gene mutation predicted a favorable prognosis and affected immunotherapy efficacy in GC [17]. DNA aneuploidy functioned as a marker associated with worse clinical outcomes in GC patients [18]. Besides, there was evidence that immune checkpoint-related genes [19], microsatellite instability (MSI) [20], and TMB [21] have predictive value for immunotherapy efficacy in GC. Therefore, we also analyzed the correlation between GI-related miRNAs and these established biomarkers.

In this study, we combined somatic mutation data and miRNA sequencing data to develop a risk signature associated with GI. Our investigation of how the signature influences diagnosis, prognosis, immune landscape, and immunotherapy responsiveness may offer insights into the relationships between miRNAs and GC. More importantly, our study indicates that these miRNAs may be exploited as predictive hallmarks and even drug targets in GC.

## 2. Materials and Methods

### 2.1. Data Collection

The Cancer Genome Atlas- (TCGA-) GC profiles in this study were retrieved from TCGA portal (https://portal.gdc.cancer.gov/), including miRNA sequencing data (446 GC samples), mRNA sequencing data (375 GC samples), clinical data (397 GC samples), and somatic mutation data (434 GC samples). The whole TCGA cohort was randomly split into two subsets. The subset used to build the risk model was designated the training set, and the other subset, which was used to validate the performance of the risk model, was designated the testing set. The characteristics of the three sets are presented in Supplementary Table 1. The GSE112264 (normal samples = 41 and GC samples = 50) and GSE54129 (normal samples = 21 and GC samples = 111) datasets used in the validation phase were downloaded from the Gene Expression Omnibus (GEO) database (https://www.ncbi.nlm.nih.gov/geo/).

### 2.2. Identification of GI-Related miRNAs

Integrating miRNA sequencing data and somatic mutation data from TCGA, GI-related miRNAs were identified using the following steps: (i) the cumulative number of somatic mutations in each patient was defined as the somatic mutation value (SMV); (ii) patients were ranked by descending SMV and clustered into two groups: the first group, with the top 25% SMVs, was characterized by genome instability (GI) and the second group, with the bottom 25% SMVs, was characterized by genome stability (GS); (iii) miRNA expression was compared between the GI and GS groups; and (iv) miRNAs that met the selection criteria (∣logfold change | = 1 and adjusted *p* < 0.05) were identified as the GI-related miRNAs, which were presented in a heatmap. The R packages “limma,” “pheatmap,” and “sparcl” were utilized to process and visualize the data. The top 40 differential miRNAs between the GS and GI groups were selected according to the absolute value of fold change, which was shown in a heatmap. Based on FunRich [22], Gene Ontology (GO) functions were applied to identify the possible biological functions of differentially expressed miRNAs. GO analysis was made up of three parts: cellular component (CC), molecular function (MF), and biological process (BP).

### 2.3. Construction of GI-Related miRNA Signature (GIMiSig)

Univariate and multivariate Cox regression analyses were performed to identify GI-related miRNAs that influenced survival. Eight miRNAs were used to establish the GIMiSig, which was used to calculate the risk score. The method to generate the risk score was as follows: risk score = (miRNA_1_ × Coef_1_ + miRNA_2_ × Coef_2_ + miRNA_3_ × Coef_3_+⋯miRNA_*n*_ × Coef_*n*_). miRNA_*n*_ represented the expression level of miRNA, and Coef_*n*_ represented the coefficient. Patients with higher risk scores (≥mean value) and lower risk scores (<mean value) were allocated to the high- and low-risk groups, respectively. Subsequently, a Kaplan-Meier analysis was used to determine the survival distributions in the high- and low-risk GC patients, and a log-rank test was used to compare the two groups, with *p* < 0.05 as the threshold. The prediction accuracy of the GIMiSig regarding survival time was assessed based on the area under the receiver operating characteristic (ROC) curve (AUC). Furthermore, we performed univariate and multivariate Cox regression analyses to identify whether the GIMiSig was an independent predictor. The hazard ratios (HRs) and *p* values were presented in forest plots. Additionally, ROC curve analysis was applied to determine the predictive performance of the GIMiSig and clinical variables regarding survival time.

### 2.4. Minimally Invasive Diagnostic Value of the GIMiSig

The expression levels of the eight miRNAs were compared between the normal and tumor groups from TCGA. Next, ROC curve analysis was performed based on the miRNA expression data of TCGA and GSE112264, which examined the performance of the GIMiSig for the diagnosis of GC.

### 2.5. Relationship between the GIMiSig and Titin (TTN) Mutation Status

Using somatic mutation data in Mutation Annotation Format, we employed the R package “maftools” to visualize the gene mutation landscape in the whole TCGA set and in the high- and low-risk groups. The ratio of wild-type to mutated TTN was compared in the high- and low-risk groups. Furthermore, GIMiSig and TTN mutation status were used to classify patients into four subgroups, and the survival distributions of the four groups were analyzed.

### 2.6. Immune Landscape Analysis and Prediction of Immunotherapy Response

First, the relationship between the GIMiSig and immune cells was determined by Spearman's correlation analysis, which is presented in Supplementary Table 2. We merged the results of multiple methods, such as TIMER, CIBERSORT, and XCELL, into a bubble diagram. The R packages “ggtext,” “ggplot2,” and “scales” were used for visualization.

Next, ESTIMATE [23] and single-sample gene set enrichment analysis (ssGSEA) [24] methods were used to evaluate the immune landscape within each risk group. ESTIMATE is an effective tool for evaluating the composition of the TME, including stromal and immune cells. Four parameters are generated by ESTIMATE: stromal score (proportion of stromal cells), immune score (proportion of immune cells), ESTIMATE score (sum of stromal and immune scores), and tumor purity (proportion of tumor cells in the tumor tissue). The analyses were completed using the R packages “estimate,” “ggplot2,” “reshape2,” and “violin.” However, ESTIMATE cannot identify specific cell subtypes. Therefore, ssGSEA was utilized to assess the differential abundance of intratumor immune cells using the R package “GSVA.” The following subtypes were included: dendritic cells (DCs), activated DCs (aDCs), immature DCs (iDCs), plasmacytoid DCs (pDCs), B cells, CD8+ T cells, macrophages, mast cells, neutrophils, natural killer (NK) cells, T helper cells, follicular helper T (Tfh) cells, type-1 T helper (Th1) cells, type-2 T helper (Th2) cells, tumor-infiltrating lymphocytes (TILs), and regulatory T cells (Tregs). We used the Wilcoxon signed-rank test to compare the immune landscape between the high- and low-risk groups.

The immune checkpoint-related gene expression levels (PDL1, CTLA4, PD1, LAG3, and TIM3) were extracted from TCGA. The MSI of each patient was determined based on somatic mutations. The aneuploidy score was retrieved from a previous study [25]. The tumor mutation burden (TMB) value was generated for each patient (by using the Perl language to analyze TCGA somatic mutation data), which was log2-transformed. The Tumor Immune Dysfunction and Exclusion (TIDE) score was calculated online (http://tide.dfci.harvard.edu//) [26]. Thereafter, the immune checkpoint-related gene levels, MSI score, aneuploidy score, TMB, and TIDE score were compared between the high- and low-risk groups.

### 2.7. Biological Significance of the GIMiSig

We carried out GSEA to elucidate the GIMiSig-related functions in GC, using the Kyoto Encyclopedia of Genes and Genomes (KEGG) as the reference. The R packages “plyr,” “ggplot2,” “grid,” and “gridExtra” were used to visualize the pathways enriched in the high- and low-risk groups.

### 2.8. Target Genes of the GIMiSig

The possible targets of the 8 miRNAs in the GIMiSig were predicted using three databases: (i) TargetScan (http://www.targetscan.org/), (ii) miRanda (http://mirdb.org/miRDB/), and (iii) miRTarBase (http://miRTarBase.mbc.nctu.edu.tw/). The overlapping results of the three platforms were regarded as putative target genes. The R package “VennDiagram” was used to generate Venn diagrams, which show the number of target genes of each miRNA according to the three databases. GO and KEGG pathway of target genes were performed using the clusterProfiler tool [27]. Based on a confidence score ≥ 0.9, target genes were selected to build a PPI network using the STRING database (http://string-db.org/). Subsequently, the network was inputted into Cytoscape, and the hub target genes were selected using CytoHubba. Finally, expression levels of the top 5 hub genes were compared between the normal and tumor groups from TCGA and GSE54129.

### 2.9. Statistical Analysis

All statistical analyses were conducted using R software (version 3.6.1). Data from different groups were compared using the Mann-Whitney-Wilcoxon test. The log-rank test was performed to assess differences in survival. *p* < 0.05 was the significance threshold.

## 3. Results

### 3.1. Different Genomic Alterations in the GS and GI Groups

**Figure 1:**
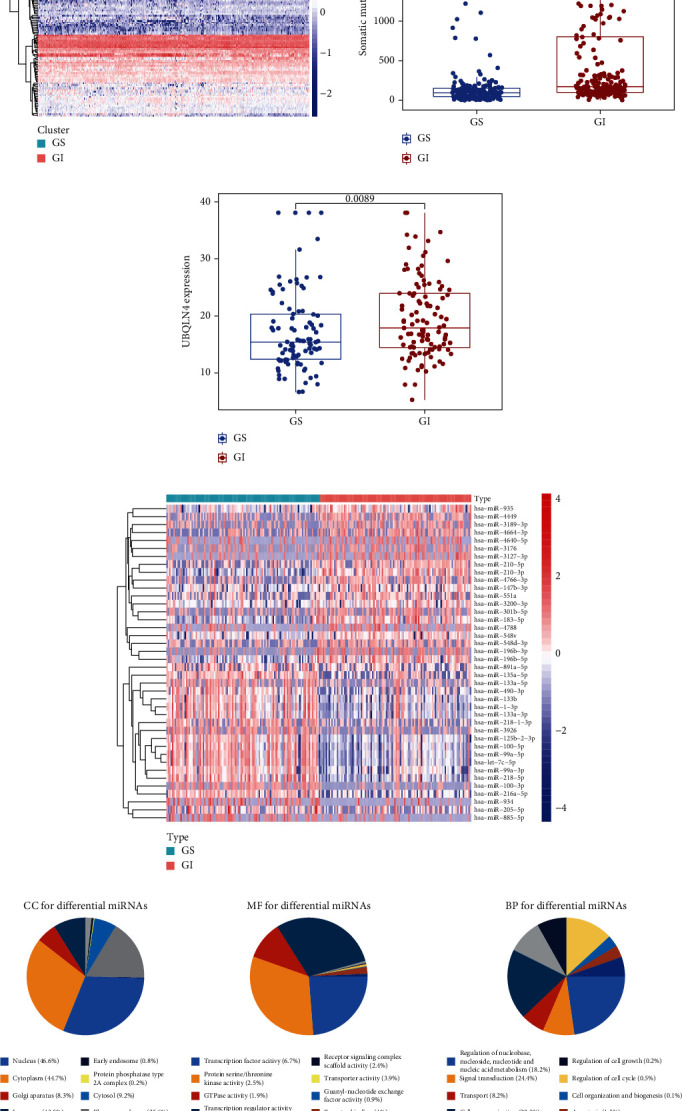
Identification of genomic features in the GS and GI groups. (a) Clustering heatmap of GS and GI patients based on differentially expressed miRNAs. The left part (red) represents the GI patients and the right part (green) represents the GS patients. (b) Somatic mutation count and (c) UBQLN4 expression were significantly elevated in the GI patients compared to the GS patients. The blue dots indicate GS patients, and the red dots indicate GI patients. (d) Heatmap of the top 40 differential miRNAs between GS and GI groups. The red represents the GI patients, and the green represents the GS patients. (e) GO analysis of differential miRNAs.

Based on the genomic profiles in the whole TCGA set, we tried to differentiate the molecular subtypes of GC. SMV (sum of somatic mutations), in descending order, was used to rank the patients. Patients with the top 25% and the bottom 25% SMVs were identified as GI patients (*n* = 108) and GS patients (*n* = 110), respectively. To obtain insights into special characteristics in patients with GI, we analyzed miRNAs, SMV, and UBQLN4 expression profiles in the two subgroups. As shown in Figure 1(a), the clustering heatmap depicts the differentially expressed miRNAs between the GI and GS patients. SMV was significantly higher in GI patients than GS patients (*p* = 5.7*e* − 16) (Figure 1(b)). UBQLN4 is a novel hallmark, contributing to the occurrence of GI [14]. Thus, we compared UBQLN4 expression between the two groups. The boxplot shows that UBQLN4 was overexpressed in the GI group compared to the GS group (*p* = 0.0089) (Figure 1(c)). These results suggested that it was reasonable to distinguish the GS and GI groups based on somatic mutations. Subsequently, the expression level of the top 40 differential miRNAs between the GS and GI groups was shown in the heatmap (Figure 1(d)). Furthermore, GO analysis was performed to explore the biological function of differentially expressed miRNAs. The results showed that these miRNAs mainly participated in nucleus (CC), transcription factor activity (MF), signal transduction (BP), regulation of nucleobase, nucleoside, nucleotide and nucleic acid metabolism (BP), and cell communication (BP) (Figure 1(e)).

### 3.2. Establishment of a GI-Related miRNA Signature (GIMiSig)

To investigate the clinical significance of GI-related miRNAs in GC, a series of bioinformatics analyses were conducted to determine whether these miRNAs correlate with the prognosis of patients. A total of 389 GC patients with clinical information were divided into the training and test sets, and the former was used to build the risk model. Using univariate and multivariate Cox regression analyses (Table 1), 8 GI-related miRNAs related to overall survival (OS) were selected to build the risk model in the training set. The expression of the 8 miRNAs in each patient and the corresponding coefficients were used to calculate a risk score for each patient as follows: Risk score = (−0.446) × miR‐125b‐5p + 0.170 × miR‐99a‐3p + 0.0815 × miR‐548v + 0.338 × miR‐100‐5p + (−0.074) × miR‐196b‐3p + (−0.061) × miR‐1275 + 0.043 × miR‐380‐3p + 0.109 × miR‐363‐3p. After calculating the risk score, patients with higher and lower risk scores compared to the mean risk score within the three sets were assigned to the high- and low-risk groups, respectively.

**Figure 2:**
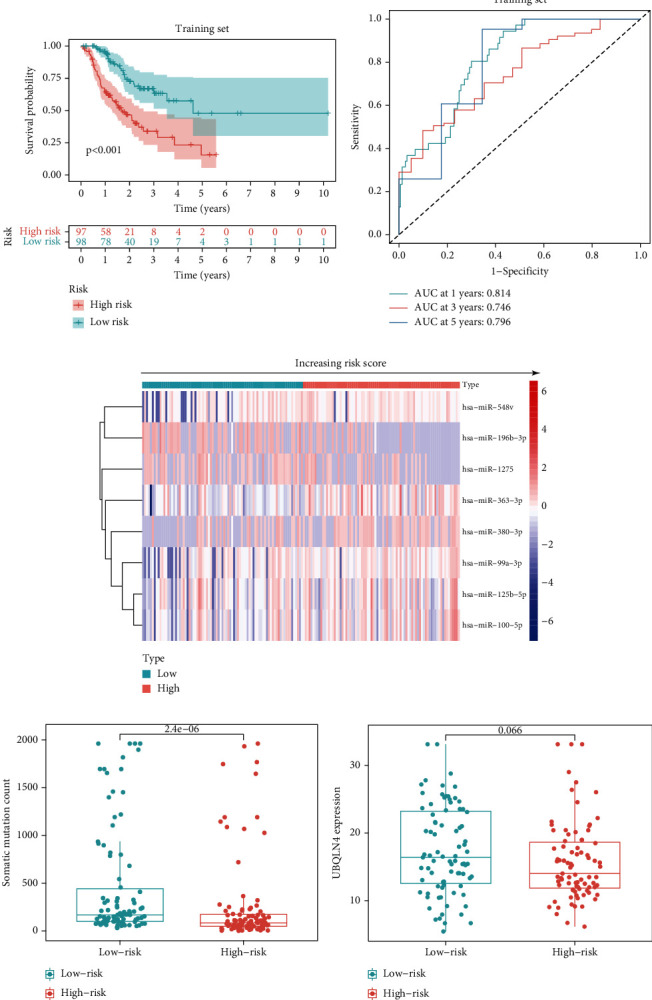
Patient characteristics in the high- and low-risk groups in the training set. (a) Survival distribution of high- and low-risk patients. (b) ROC curves predicting 1-, 3-, and 5-year survival rates. (c) Expression of the 8 miRNAs changed with increasing risk score. Comparisons of somatic mutation count (d) and UBQLN4 expression (e) in the high- and low-risk groups.

When the GIMiSig was applied to the training set, the survival data suggested that high-risk patients exhibited shorter overall survival than low-risk patients (*p* < 0.01) (Figure 2(a)). AUC at 1, 3, and 5 years was 0.814, 0.746, and 0.796, respectively (Figure 2(b)). Expression levels of the 8 miRNAs in the high- and low-risk patients were showed in the heatmap (Figure 2(c)). Moreover, low-risk patients had higher SMV (*p* = 2.4*e* − 06) (Figure 2(d)). No significant difference was observed in UBQLN4 expression between the high- and low-risk groups in the training set (*p* = 0.066) (Figure 2(e)).

**Figure 3:**
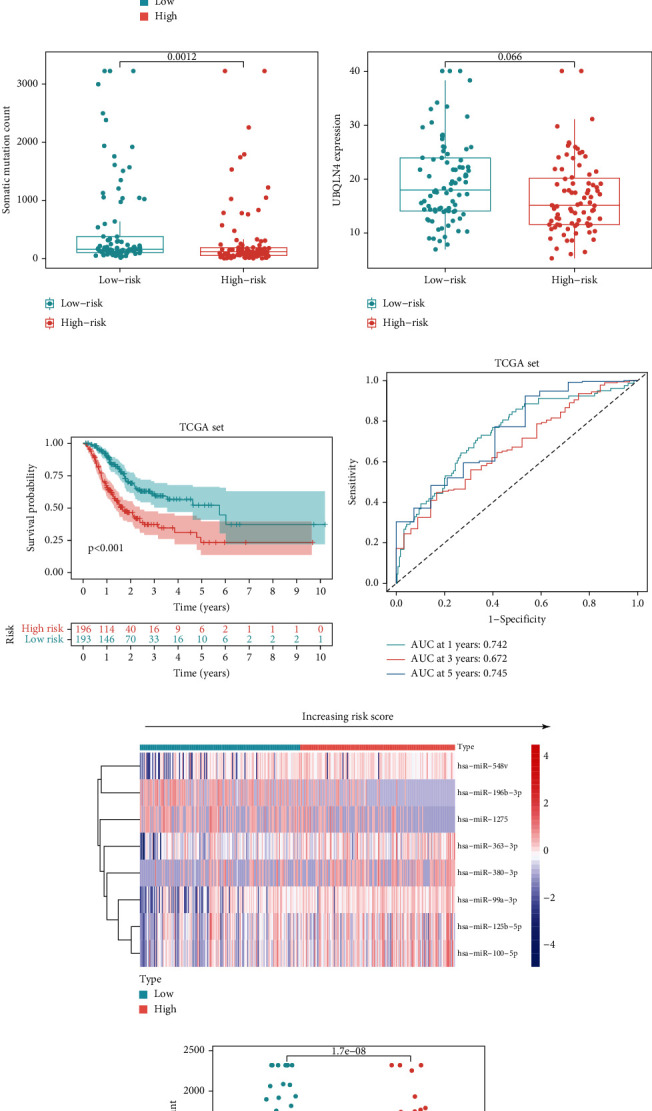
Patient characteristics in the high- and low-risk groups in the testing and whole sets. (a, f) Survival distribution of high- and low-risk patients. (b, g) ROC curves predicting 1-, 3-, and 5-year survival rates. (c, h) Expression of the 8 miRNAs changed with increasing risk score. Comparisons of somatic mutation count and UBQLN4 expression in the high- and low-risk groups in the (d, e) testing and (i, j) whole TCGA sets.

To confirm the stability of the GIMiSig, we examined its performance in the testing and whole TCGA sets. Similarly, high-risk patients had a poorer prognosis in the testing (*p* = 0.007) and whole TCGA (*p* < 0.001) sets (Figures 3(a) and 3(f)). The ROC curves for the GIMiSig also indicated good accuracy in the testing and whole TCGA sets (Figures 3(b) and 3(g)). Distribution of miRNA expression levels changed with increasing risk scores in the test and whole sets (Figures 3(c) and 3(h)). As shown in the boxplot, higher SMV (*p* < 0.01) and UBQLN4 overexpression (*p* < 0.05) were observed in low-risk patients in the testing and whole sets (Figures 3(d), 3(e), 3(i), and 3(j)). These results indicated that the GIMiSig could stratify GC patients into two groups differing in prognosis, SMV, and UBQLN4 expression.

### 3.3. Evaluation of Independent Prognostic Factors

**Figure 4:**
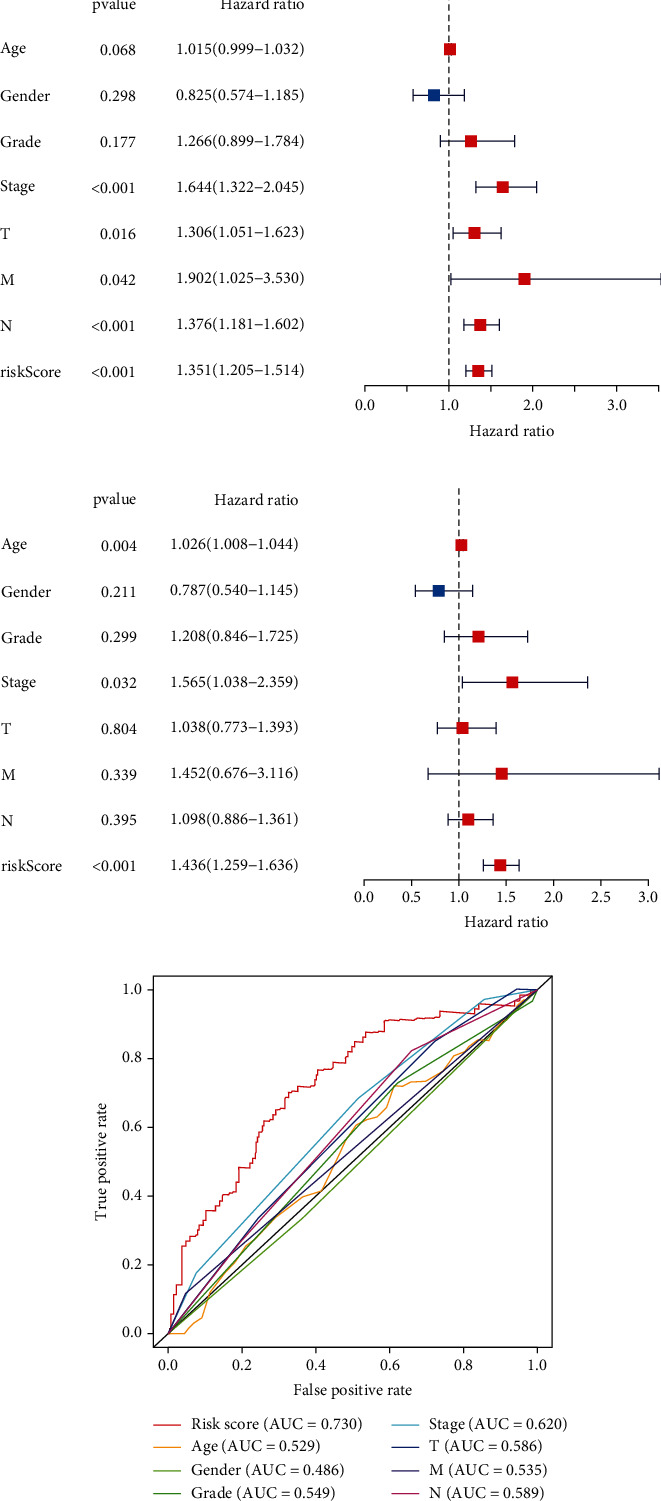
Independent risk factor analysis in the whole TCGA set. (a) Univariate and (b) multivariate Cox regression analyses were performed to calculate the hazard ratio (HR) and *p* value for each parameter. (c) AUC values of risk score, age, gender, grade, and TNM stage.

The risk score generated by the GIMiSig was identified as a prognostic factor by univariate analysis (*p* < 0.001, HR = 1.351, 95% CI 1.205–1.514) (Figure 4(a)) and as a factor that was independent of the other clinical features by multivariate analysis (*p* < 0.001, HR = 1.436, 95% CI 1.259–1.636) (Figure 4(b)). More importantly, the risk score yielded the highest AUC value (0.730) compared to other parameters, such as age (0.529) and grade (0.549), which indicated that the GIMiSig is an effective tool for risk assessment of GC patients (Figure 4(c)).

### 3.4. Survival Analysis of GC Subgroups

**Figure 5:**
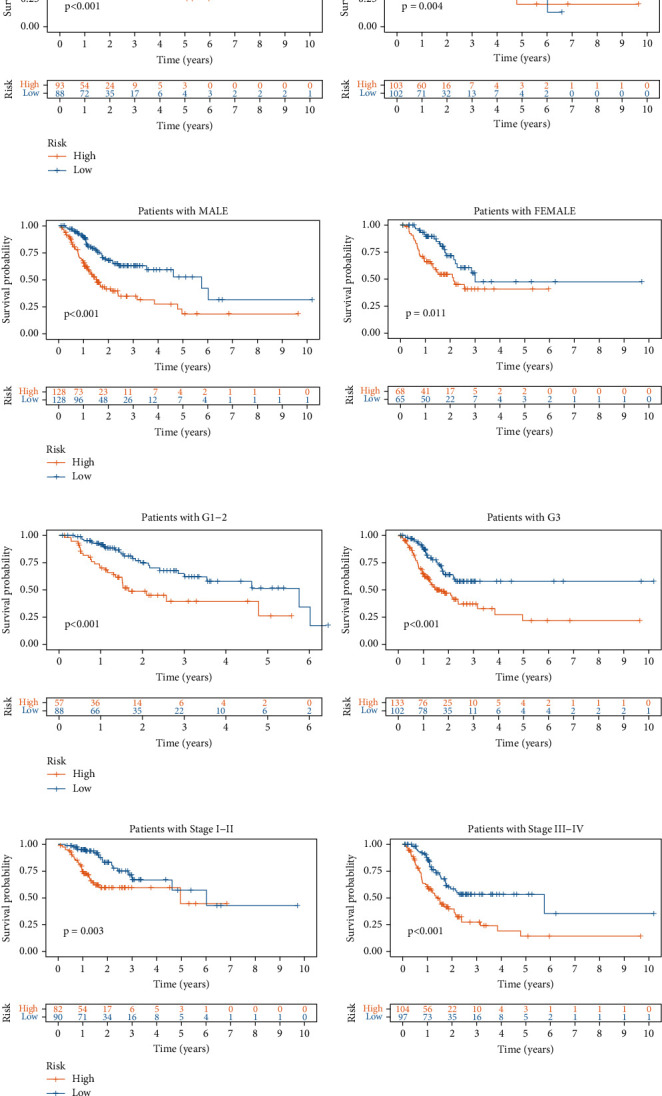
Survival analysis in high- and low-risk subgroups stratified by (a) age, (b) gender, (c) grade, and (d) stage.

We then examined the survival time of patients in the high- and low-risk subgroups stratified by age, gender, stage, and grade. Low-risk patients had better survival time than high-risk patients in the young (age < 65 years, *p* < 0.001) and old (age ≥ 65 years, *p* = 0.004) subgroups (Figure 5(a)), female (*p* < 0.001) and male (*p* = 0.011) subgroups (Figure 5(b)), stage I–II (*p* = 0.003) and stage III–IV (*p* < 0.001) (Figure 5(c)) subgroups, and grade 1–2 (*p* < 0.001) and grade 3 (*p* < 0.001) subgroups (Figure 5(d)).

### 3.5. Minimally Invasive Diagnostic Value of the GIMiSig

**Figure 6:**
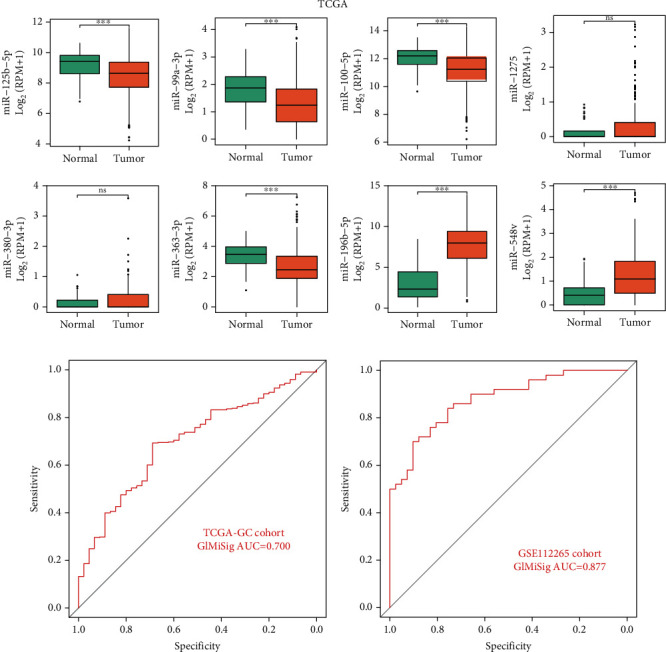
Diagnostic value of the GIMiSig. (a) Expression levels of the 8 miRNAs between the normal and tumor groups in TCGA. AUC values of the GIMiSig to diagnose GC patients in TCGA cohort (b) and GSE112264 cohort (c). Orange indicates the tumor group, and green indicates the normal group. ^∗^*p* < 0.05, ^∗∗^*p* < 0.01, and ^∗∗∗^*p* < 0.001.

In addition, we assessed the minimally invasive diagnostic value of the GIMiSig. First, expression levels of the eight miRNAs were compared between the normal and tumor groups from TCGA. We found that miR-125b-5p (*p* < 0.001), miR-99a-3p (*p* < 0.001), miR-100-5p (*p* < 0.001), miR-548v (*p* < 0.001), and miR-363-3p (*p* < 0.001) were downregulated in tumors compared with that in normal control. On the contrary, miR-196b-3p (*p* < 0.001) was highly expressed in tumors than that in normal control. There was no significant difference of miR-380-3p and miR-1275 between normal and tumor (Figure 6(a)). The differential expression of these miRNAs indicated that the GIMiSig may have the potential for early diagnosis of GC. Next, we evaluated whether the GIMiSig could differentiate GC from normal control in TCGA and GSE112264. The results revealed that the AUC of TCGA and GSE112264 cohort were 0.700 (Figure 6(b)) and 0.877 (Figure 6(c)), indicating the GIMiSig was able to detect GC patients accurately.

### 3.6. Relationship between the GIMiSig and TTN Mutation Status

**Figure 7:**
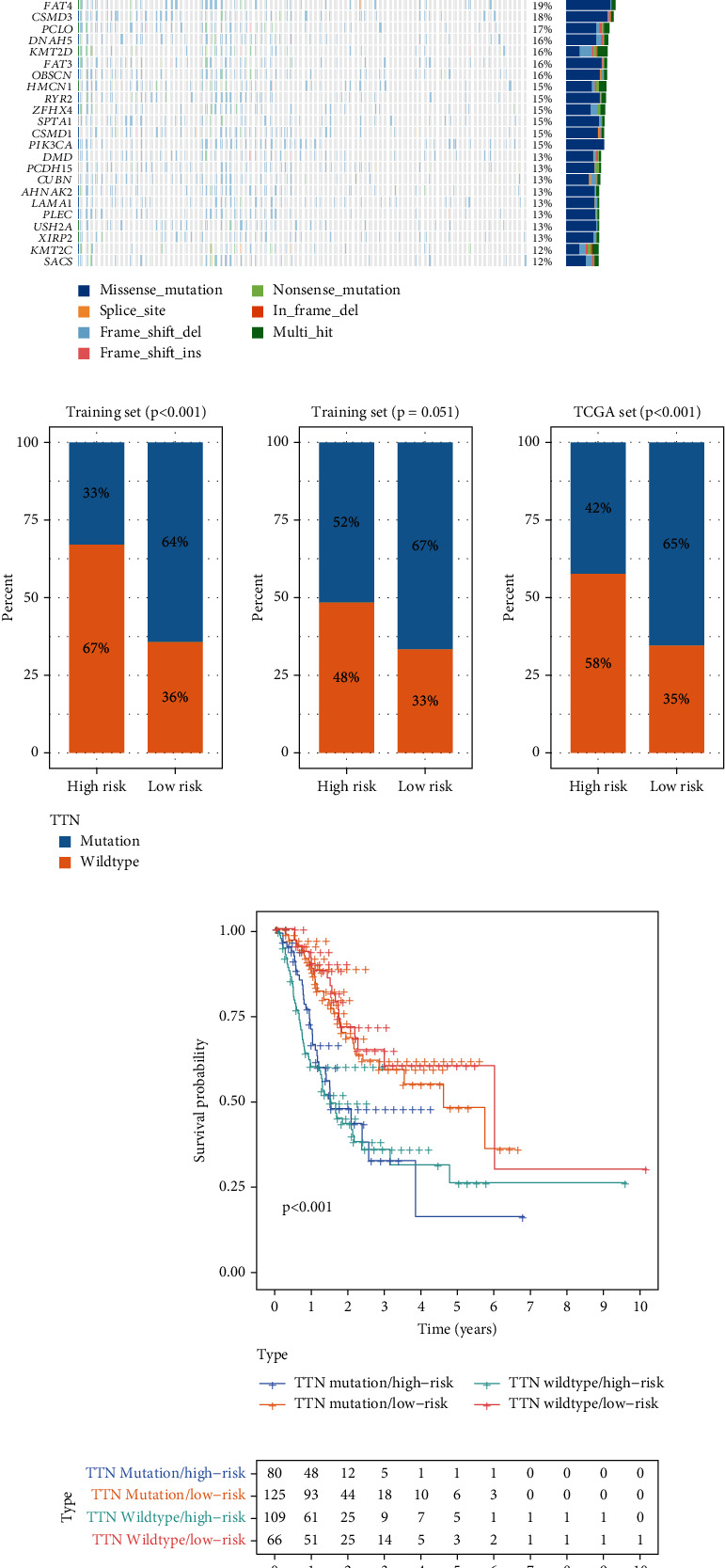
Relationship between the GIMiSig and TTN mutation status. (a) Overview of gene mutations in GC patients from TCGA. (b) Distribution of wild and mutated TTN in the high- and low-risk groups in the training, testing, and whole sets. (c) Survival curves for patients stratified by TTN mutation status and GIMiSig.

As shown in the waterfall plot (Figure 7(a)), TTN mutation accounted for 48% of all gene variants in the whole TCGA set. Additionally, the proportion of mutated TTN was higher than the proportions of other gene mutations in both the high-risk (38%) and low-risk (58%) groups (Supplementary Figure 1). Thus, we next examined the distribution of wild-type and mutated TTN in the high- and low-risk groups. The results suggested that low-risk patients had more frequent TTN mutation than high-risk patients in the three sets (Figure 7(b)). In the training set, TTN mutation was detected in 30% of high-risk patients and 64% of low-risk patients (*p* < 0.001). Although there was no significant association of TTN mutation status with risk in the test set, there was a trend toward more frequent TTN mutation in the low-risk patients (*p* = 0.051). In the whole TCGA set, TTN mutation was more common in the low-risk group (65%) than the high-risk group (42%) (*p* < 0.001). We further assessed whether the GIMiSig could predict survival better than TTN mutation status. Using TTN mutation status, patients were classified into the TTN mutation and TTN wild-type groups. There were no significant differences in OS between TTN mutation and wild-type TTN among the high-risk patients or between TTN mutation and wild-type TTN among the low-risk patients (*p* > 0.05) (Figure 7(c)). When the GIMiSig was used to divide patients into the high- and low-risk groups, we observed significant differences in OS between the high- and low-risk groups among patients with TTN mutation or with wild-type TTN (*p* < 0.001, Figure 7(c)).

### 3.7. Relationship between the GIMiSig and Immune Landscape

**Figure 8:**
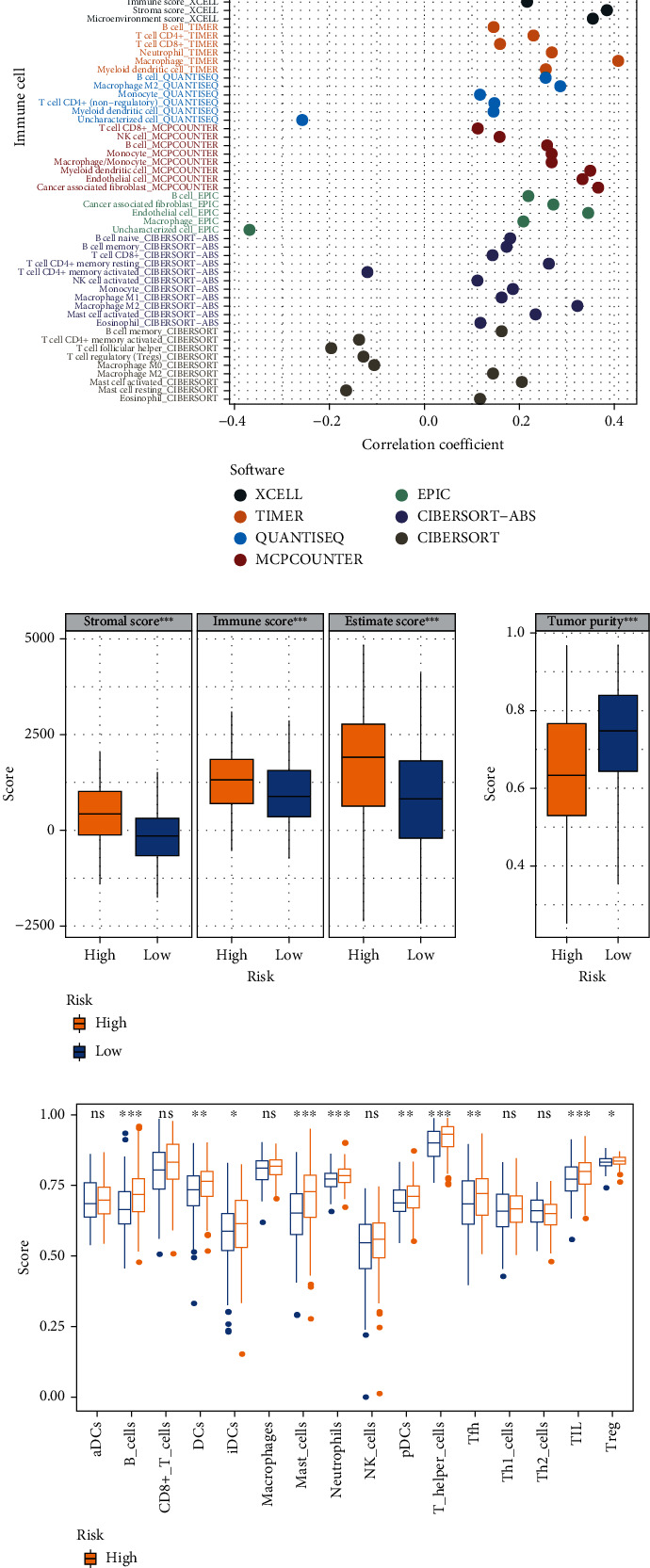
Immune landscape in the high- and low-risk groups. (a) Correlation analysis between the GIMiSig and various intratumor cell subpopulations. (b) Evaluation of immune landscape in the high- and low-risk groups based on ESTIMATE. (c) Boxplots showing distribution of immune cells in the high- and low-risk groups using the ssGSEA algorithm. Yellow indicates the high-risk group, and blue indicates the low-risk group. ^∗^*p* < 0.05, ^∗∗^*p* < 0.01, and ^∗∗∗^*p* < 0.001.

Accumulating evidence has shown that the TME greatly impacts tumor occurrence and responses to immune-targeting strategies. Multiple methods were utilized to evaluate the association of the GIMiSig with the TME. First, correlation analysis was conducted between the GIMiSig and various intratumor cell subpopulations. The results revealed that high-risk patients exhibited positive associations with CD8+ T cells, fibroblasts, B cells, and macrophages while they had negative associations with activated CD4+ T cells, T helper cells, and Treg cells (Figure 8(a)). Next, the ESTIMATE and ssGSEA algorithms were used to evaluate immune characteristics in the high- and low-risk groups. ESTIMATE was applied to calculate the stromal, immune, and ESTIMATE scores for each GC patient. Interestingly, compared to the low-risk patients, the high-risk patients had higher stromal, immune, and ESTIMATE scores but lower tumor purity (*p* < 0.001) (Figure 8(b)). ssGSEA was performed to investigate the relationships between GIMiSig and 14 intratumoral immune cells. The high-risk patients had higher proportions of B cells (*p* < 0.001), DCs (*p* < 0.01), iDCs (*p* < 0.05), mast cells (*p* < 0.01), neutrophils (*p* < 0.001), pDCs (*p* < 0.01), T helper cells (*p* < 0.01), follicular helper T (Tfh) cells (*p* < 0.01), TILs (*p* < 0.001), and Treg cells (*p* < 0.05) (Figure 8(c)). In aggregate, these data suggested that the GIMiSig may influence the immune microenvironment.

### 3.8. Prediction of Immunotherapy Responsiveness

**Figure 9:**
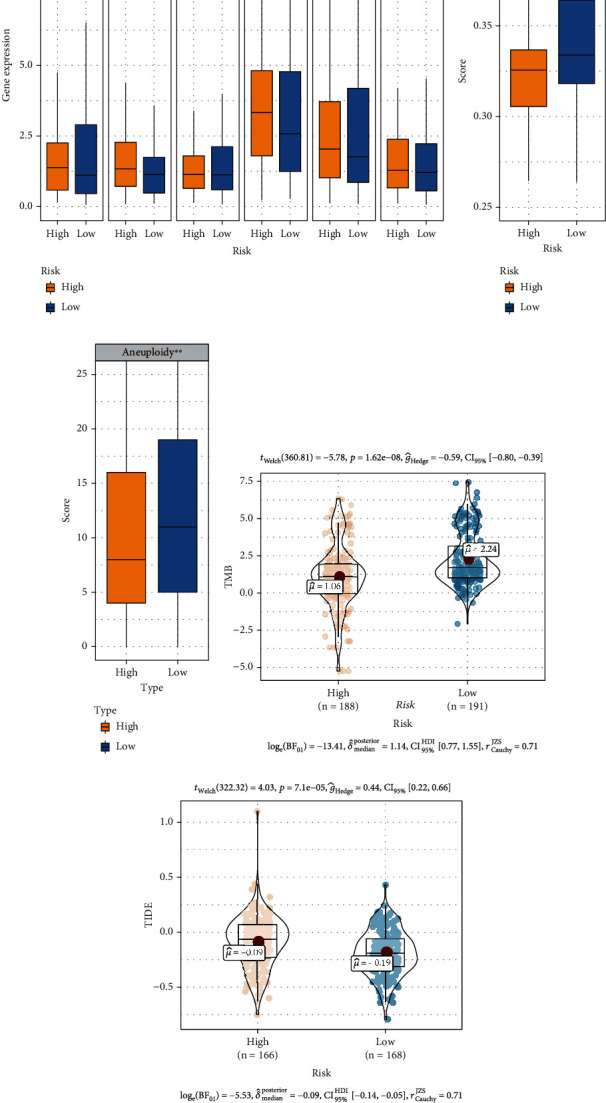
Prediction of immunotherapy responsiveness. (a) Relationships between the GIMiSig and immune checkpoint-related gene expression levels. Comparison of (b) microsatellite instability (MSI) score, (c) aneuploidy score, (d) tumor mutation burden (TMB), and (e) Tumor Immune Dysfunction and Exclusion (TIDE) score in the high- and low-risk groups. ^∗^*p* < 0.05, ^∗∗^*p* < 0.01, and ^∗∗∗^*p* < 0.001.

Immune checkpoint-related gene expression levels, MSI score, aneuploidy score, TMB, and TIDE score were used to assess the potential clinical efficacy of immunotherapy in the high- and low-risk groups from TCGA. We found that PD-L2 (*p* < 0.01) and TIM3 (*p* < 0.05) expression was significantly higher in the high-risk group than in the low-risk group (Figure 9(a)). The GIMiSig had no significant influence on other immune checkpoint-related genes, although a trend toward elevated CTLA-4 (*p* > 0.05) expression was observed in high-risk patients. Additionally, the MSI score was lower in low-risk patients than high-risk patients (*p* < 0.001) (Figure 9(b)). As for the aneuploidy score, it was higher in low-risk patients than high-risk patients (*p* < 0.01) (Figure 9(c)). Patients with high TMB are more likely to trigger immune responses by presenting more neoantigens, so they benefit more from ICI therapy. Tumor cells in patients with high TIDE scores are more likely to escape from the surveillance of the immune system, resulting in insensitivity to ICI therapy. In this study, high-risk patients had lower TMB (*p* = 1.62*e* − 08) (Figure 9(d)) and higher TIDE score (*p* = 7.4*e* − 05) (Figure 9(e)) compared to low-risk patients, suggesting that high-risk patients may have unfavorable responses to immunotherapy.

### 3.9. GSEA

**Figure 10:**
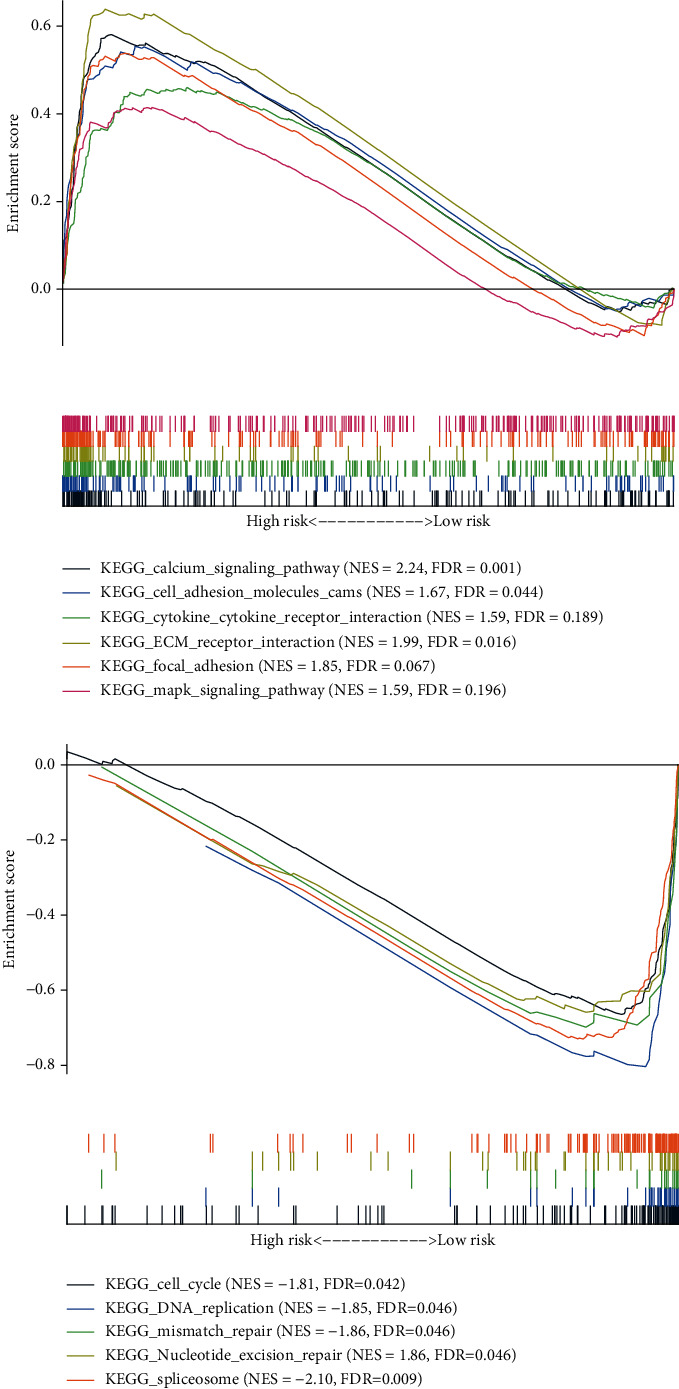
GSEA of the whole TCGA set: (a) enriched pathways in high-risk patients; (b) enriched pathways in low-risk patients.

To elucidate the link between the GIMiSig and its functional properties in GC, GSEA was used to detect the GIMiSig-related pathways with the criterion of normalized *p* value (norm *p*) < 0.05 and False Discovery Rate (FDR) < 0.25. The results showed that several cancer-associated and immune response pathways were discovered in the high-risk patients, such as cell adhesion molecules, cytokine-cytokine receptor interaction, and the MAPK signaling pathway (Figure 10(a)). Additionally, several GI-related pathways were enriched in the low-risk patients, including cell cycle, nucleotide excision repair, and mismatch repair (Figure 10(b)). This might explain why higher SMV and UBQLN4 overexpression was detected in low-risk patients. Taken together, these results indicated potential roles of the 8 aberrantly expressed miRNAs in the occurrence and progression of GC: (i) contributing to tumor metastasis via cell adhesion molecules, (ii) altering the immune microenvironment based on cytokine release, and (iii) disrupting gene repair.

### 3.10. Target Genes of the GIMiSig

**Figure 11:**
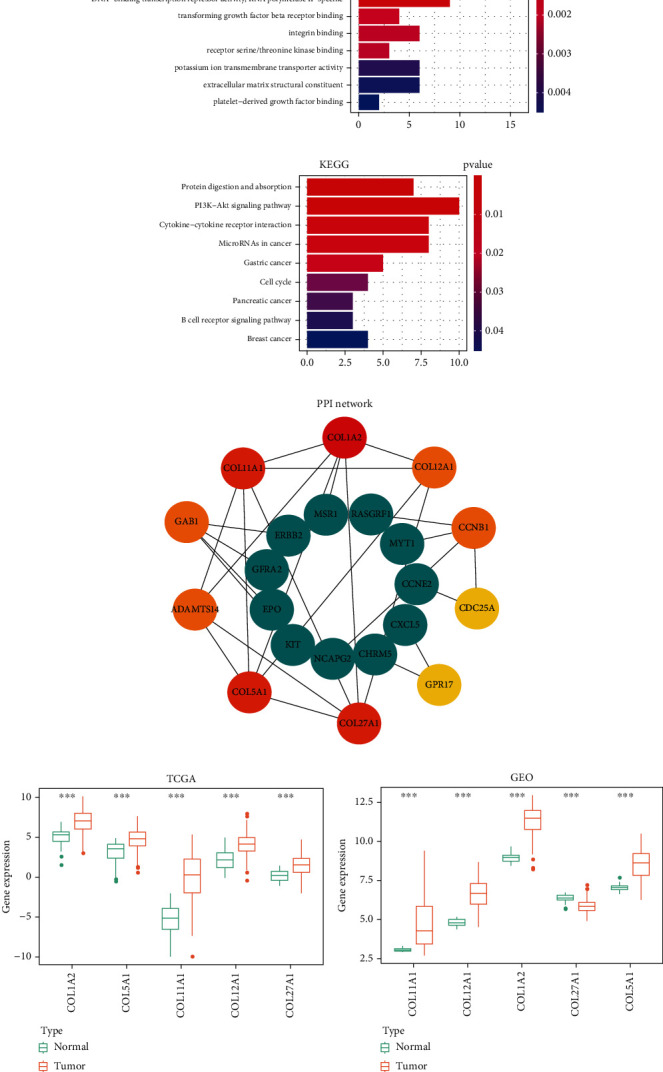
Target genes of the GIMiSig. (a) GO and (b) KEGG analyses of target genes. (c) PPI network of hub genes. The top 10 hub genes are indicated in red, orange, and yellow. (d) Expression of the 5 genes was compared between normal and tumor groups from TCGA and (e) GSE54129. Orange indicates the tumor group, and green indicates the normal group. ^∗^*p* < 0.05, ^∗∗^*p* < 0.01, and ^∗∗∗^*p* < 0.001.

To ascertain the downstream targets of the 8 miRNAs, we predicted the target genes using three public databases (TargetScan, miRanda, and miRTarBase) and presented the overlapping results (target genes shared across all three databases) in Venn diagrams. There were 31, 12, 24, 135, 106, 2, and 59 target genes of miR-548v, miR-100-5p, miR-380-3p, miR-363-3p, miR-125b-5p, miR-196b-3p, and miR-1275, respectively (Supplementary Figure 2). As there were no overlapping target genes for miR-99a-3p, only the remaining 7 miRNAs were retained to construct the miRNA-target regulatory network. After obtaining the target gene list, GO and KEGG analyses were conducted to identify enriched biological processes of target genes. We found that target genes may play roles in several GI-related biological pathways, such as DNA-binding transcription activator activity, RNA polymerase II-specific, chromatin DNA binding, and cell cycle (Figures 11(a) and 11(b)). Next, the interactions among target genes were investigated using a PPI network analysis. The network was inputted into Cytoscape, and hub target genes were selected by CytoHubba. Finally, based on the connectivity degree, the top 5 hub target genes were as follows: COL1A2 (degree = 6), COL5A1 (degree = 5), COL11A1 (degree = 5), COL27A1 (degree = 5), and COL12A1 (degree = 4) (Figure 11(c)). Thus, our analyses showed that the 8 miRNAs in the GIMiSig may target these hub genes to affect biological processes in GC. Besides, all the top 5 hub genes had significantly higher expression in GC compared with that in normal samples from TCGA (*p* < 0.001) (Figure 11(d)) and GSE54129 (*p* < 0.001) (Figure 11(e)).

## 4. Discussion

To the best of our knowledge, this is the first study to establish a GIMiSig and explore its predictive value and biological function in GC. The GIMiSig consisted of 8 GI-related miRNAs (miR-99a-3p, miR-548v, miR-100-5p, miR-380-3p, miR-363-3p, miR-125b-5p, miR-196b-3p, and miR-1275). The risk score generated by the GIMiSig was utilized to distinguish the high- and low-risk groups. The GIMiSig exhibited better predictive performance than current clinical classification methods, with the survival analysis and ROC curves showing that the GIMiSig was an effective tool for predicting survival. Of note, the GIMiSig was associated with frequency of TTN mutation, but it predicted the prognosis more accurately than TTN mutation status. Furthermore, the GIMiSig exerted an influence on the immune landscape and was also capable of predicting immunotherapy responses. Finally, GSEA revealed that the GIMiSig was closely related to cancer-associated, immune response, and GI-related pathways.

Six miRNAs (miR-99a, miR-100-5p, miR-363-3p, miR-1275, miR-196b-3p, and miR-125b-5p) in the GIMiSig have previously been reported in GC. miR-99a and miR-100-5p were regarded as tumor suppressors in GC due to their function of inhibiting cell proliferation [28, 29]. miR-363-3p expression was downregulated in GC, and its expression was associated with invasion and lymphatic metastasis [30]. miR-1275 prevented metastasis and was associated with survival time in GC [31]. Overexpression of miR-196b-3p was discovered in GC samples and affected the epithelial-mesenchymal transition [32]. Upregulation of miR-125b-5p was observed in GC and led to invasion and metastasis by two possible pathways: (i) targeting STARD13 and NEU1 mRNAs and (ii) targeting the PPP1CA-Rb axis [33, 34]. Specifically, elevated miR-125b-5p resulted in progression of GC and poor response to trastuzumab [35]. Though the remaining 2 miRNAs (miR-548v and miR-380-3p) in the GIMiSig have not been reported in GC, they have been identified as potential biomarkers in multiple cancers, including endometrial cancer [36], lung adenocarcinoma [37], breast cancer [38], and neuroblastoma [39].

Interestingly, high somatic mutation and UBQLN4 expression, which indicated high GI, were observed in low-risk patients, who had longer survival time in our study. In these cases, more mutations of tumor driver genes occurred, but more neoantigens would also be presented to trigger immune response [40]. Additionally, UBQLN4 can suppress the progression of GC by preventing tumor cell proliferation [15]. This may partly explain why a high somatic mutation number and UBQLN4 expression were detected in patients with longer survival time. TTN was known as a gene associated with familial hypertrophic cardiomyopathy [41]. Yang et al. found that TTN mutation was effective for predicting prognosis, TMB, and immunotherapy response in GC [17]. Thus, we investigated the relationship between the GIMiSig and TTN mutation status. Our study suggested that the high- and low-risk groups (based on the GIMiSig) exhibited a significant difference in the frequency of TTN mutation, indicating that the GIMiSig was able to capture the TTN mutation status. More importantly, when the TTN gene was used to classify patients into wild-type and mutation groups, there were no significant differences in OS between TTN mutation and wild-type TTN among the high-risk patients or between TTN mutation and wild-type TTN among the low-risk patients. Conversely, the GIMiSig was capable of distinguishing distinct clinical prognoses among patients with TTN mutation and among patients without TTN mutation. This suggests that the GIMiSig is a better predictor of prognosis in GC than TTN mutation status.

In our study, the two groups divided based on the GIMiSig exhibited distinct immune landscapes. After extensively reviewing the literature, we found that miR-99a, miR-100-5p, miR-125b, and miR-363-3p in the GIMiSig were involved in the regulation of immune status, mainly through influencing the function of immune cells. Jaiswal et al. showed that miR-99a downregulated TNF-*α*, resulting in an increased ratio of M2/M1 macrophages [42]. Additionally, miR-99a promoted Treg differentiation and decreased cytotoxic T lymphocytes [43]. Thus, miR-99a impaired the ability of immune cells to kill tumor cells. Similarly, miR-100-5p expression was also associated with macrophage polarization and Treg differentiation [44, 45]. High miR-125b expression could disturb B cell development and suppress effector T cell bioactivities, while enhancing the proinflammatory nature of M1 macrophages [46]. Of note, due to the crucial roles of miR-125b in the immune system, it demonstrated a promising ability to predict immunotherapy response in several tumors, such as non-small-cell lung cancer (NSCLC) [47], prostate cancer [48], and colorectal cancer [49]. As for miR-363-3p, it affected several crucial transcription factors that regulated Th17 cell differentiation [50]. Combining previous studies and our findings, we speculate that the GIMiSig is suitable for evaluating the immune landscape in GC.

Another highlight of our study is the associations of the GIMiSig with known immunotherapy responsiveness biomarkers (PD-L1, MSI score, aneuploidy score, TMB, and TIDE score). Although ICIs have exhibited encouraging clinical trial results in several tumors [49, 51], their efficacy is not satisfactory in GC. Evidence shows that ICIs are only beneficial for specific GC subtypes, such as those involving deficiency mismatch repair [52]. Therefore, predictive biomarkers are urgently needed to distinguish patients that could benefit from immunotherapy. As PD-L1-positive patients are more sensitive to ICIs, PD-L1 is regarded as an index of immunotherapy efficacy [52]. High MSI is also associated with better immunotherapy responses in GC [53] while a high aneuploidy score correlates with reduced response to immunotherapy [54]. TMB reflects the quantity of somatic mutations and is associated with ICI responses in GC, and it can serve as a biomarker for predicting immunotherapy efficacy [55]. Patients with high TMB tend to obtain more clinical benefits from ICI treatment [56]. The TIDE score is a novel algorithm based on tumor immune escape, which provides clues for selecting patients that are suitable for ICI treatment. In this study, there was no significant difference in PD-L1 between the high- and low-risk patients. However, the low-risk patients had higher MSI scores, aneuploidy scores, and TMB, along with lower TIDE scores. Thus, we inferred that high-risk patients were more likely to benefit from immunotherapy. Moreover, these findings indicate that the GIMiSig could be exploited as a biomarker of immunotherapy responses.

There are several limitations to our study. First, the mechanisms underlying the associations between the GIMiSig and immune landscape, TMB, and ICI responses remain unclear. Second, the data on the cohort used in our study did not include data on GC patients taking immunotherapy, so the ability of the GIMiSig to predict immunotherapy efficacy remains to be elucidated. Third, biology experiments and clinical data are expected to testify to the performance of this GIMiSig before clinical application.

## 5. Conclusions

In conclusion, we constructed a GIMiSig in GC based on GI-related miRNAs and investigated its prognostic value and potential function. Our study strongly suggests that the GIMiSig not only is of significance for predicting the prognosis and immune landscape of GC but also provides new insights into future immunotherapy targets.

## Figures and Tables

**Table 1:** Multivariate Cox regression analyses in the training set.

id	coef	HR	HR.95L	HR.95H	*p* value
miR-125b-5p	-0.446	0.640	0.409	1.000	0.050
miR-99a-3p	0.110	1.116	1.000	1.246	0.049
miR-548v	0.082	1.085	1.008	1.168	0.030
miR-100-5p	0.338	1.402	0.934	2.105	0.103
miR-196b-3p	-0.074	0.929	0.888	0.971	0.001
miR-1275	-0.061	0.940	0.900	0.982	0.006
miR-380-3p	0.043	1.044	0.995	1.096	0.080
miR-363-3p	0.109	1.116	0.969	1.285	0.129

## Data Availability

The data of this study are available in TCGA (https://portal.gdc.cancer.gov/) and GEO database (https://www.ncbi.nlm.nih.gov/geo/).

## Supplementary Materials

Supplementary Materials Supplementary Table 1: clinical information of three sets. Supplementary Table 2: correlation analysis between the GIMiSig and immune cells. Supplementary Figure 1: gene mutation landscapes of high-risk (A) and low-risk (B) groups. Supplementary Figure 2: Venn diagrams showing overlapping target genes according to TargetScan, MiRanda, and miRTarBase.
