# Supplementary material for: Genome Instability-Related miRNAs Predict Survival, Immune Landscape, and Immunotherapy Responses in Gastric Cancer
Source: J Immunol Res. 2021 Nov 1;2021:2048833. doi: 10.1155/2021/2048833 (PMC8575650; doi:10.1155/2021/2048833)
Supplement: Supplementary Materials — Supplementary Table 1: clinical information of three sets. Supplementary Table 2: correlation analysis between the GIMiSig and immune cells. Supplementary Figure 1: gene mutation landscapes of high-risk (A) and low-risk (B) groups. Supplementary Figure 2: Venn diagrams showing overlapping target genes according to TargetScan, MiRanda, and miRTarBase. [file 2048833.f1.docx]

Supplementary Material

Supplementary Table 1. Clinical information of three sets

| Covariates |  | TCGA set | Train set | Test set | P-value |
| --- | --- | --- | --- | --- | --- |
|  |  |  |  |  |  |
| Age | <=65 | 181(46.53%) | 89(45.64%) | 92(47.42%) |  |
|  | >65 | 205(52.7%) | 105(53.85%) | 100(51.55%) | 0.764 |
|  | unknown | 3(0.77%) | 1(0.51%) | 2(1.03%) |  |
| Gender | Female | 133(34.19%) | 65(33.33%) | 68(35.05%) | 0.802 |
|  | Male | 256(65.81%) | 130(66.67%) | 126(64.95%) |  |
| Grade | G1-G2 | 145(37.28%) | 65(33.33%) | 80(41.24%) |  |
|  | G3 | 235(60.41%) | 126(64.62%) | 109(56.19%) | 0.120 |
|  | unknown | 9(2.31%) | 4(2.05%) | 5(2.58%) |  |
| Stage | Stage I-II | 172(44.22%) | 90(46.15%) | 82(42.27%) |  |
|  | Stage III-IV | 201(51.67%) | 94(48.21%) | 107(55.15%) | 0.334 |
|  | unknown | 16(4.11%) | 11(5.64%) | 5(2.58%) |  |
| T | T1-2 | 103(26.48%) | 50(25.64%) | 53(27.32%) |  |
|  | T3-4 | 281(72.24%) | 142(72.82%) | 139(71.65%) | 0.818 |
|  | unknown | 5(1.29%) | 3(1.54%) | 2(1.03%) |  |
| M | M0 | 350(89.97%) | 179(91.79%) | 171(88.14%) |  |
|  | M1 | 25(6.43%) | 8(4.1%) | 17(8.76%) | 0.101 |
|  | unknown | 14(3.6%) | 8(4.1%) | 6(3.09%) |  |
| N | N0 | 115(29.56%) | 63(32.31%) | 52(26.8%) |  |
|  | N1-3 | 263(67.61%) | 126(64.62%) | 137(70.62%) | 0.264 |
|  | unknown | 11(2.83%) | 6(3.08%) | 5(2.58%) |  |

Supplementary Table 2 Correlation analysis between the GIMiSig and immune cells

| Cell subtypes | Cor | P-value |
| --- | --- | --- |
|  |  |  |
| B cell_TIMER | 0.143 | p < 0.01 |
| T cell CD4+_TIMER | 0.227 | p < 0.001 |
| T cell CD8+_TIMER | 0.156 | p < 0.01 |
| Neutrophil_TIMER | 0.265 | p < 0.001 |
| Macrophage_TIMER | 0.405 | p < 0.001 |
| Myeloid dendritic cell_TIMER | 0.253 | p < 0.001 |
| B cell memory_CIBERSORT | 0.160 | p < 0.01 |
| T cell CD4+ memory activated_CIBERSORT | -0.141 | p < 0.01 |
| T cell follicular helper_CIBERSORT | -0.199 | p < 0.001 |
| T cell regulatory (Tregs)_CIBERSORT | -0.131 | p < 0.05 |
| Macrophage M0_CIBERSORT | -0.109 | p < 0.05 |
| Macrophage M2_CIBERSORT | 0.141 | p < 0.01 |
| Mast cell activated_CIBERSORT | 0.202 | p < 0.001 |
| Mast cell resting_CIBERSORT | -0.168 | p < 0.01 |
| Eosinophil_CIBERSORT | 0.114 | p < 0.05 |
| B cell naive_CIBERSORT-ABS | 0.178 | p < 0.001 |
| B cell memory_CIBERSORT-ABS | 0.170 | p < 0.01 |
| T cell CD8+_CIBERSORT-ABS | 0.140 | p < 0.01 |
| T cell CD4+ memory resting_CIBERSORT-ABS  T cell CD4+ memory activated_CIBERSORT-ABS  NK cell activated_CIBERSORT-ABS | 0.260  -0.123  0.109 | p < 0.001  p < 0.05  p < 0.05 |
| Monocyte_CIBERSORT-ABS | 0.183 | p < 0.001 |
| Macrophage M1_CIBERSORT-ABS | 0.160 | p < 0.01 |
| Macrophage M2_CIBERSORT-ABS | 0.319 | p < 0.001 |
| Mast cell activated_CIBERSORT-ABS | 0.231 | p < 0.001 |
| Eosinophil_CIBERSORT-ABS | 0.115 | p < 0.05 |
| B cell_QUANTISEQ | 0.252 | p < 0.001 |
| Macrophage M2_QUANTISEQ | 0.283 | p < 0.001 |
| Monocyte_QUANTISEQ | 0.114 | p < 0.05 |
| T cell CD4+ (non-regulatory)_QUANTISEQ | 0.144 | p < 0.01 |
| Myeloid dendritic cell_QUANTISEQ | 0.143 | p < 0.01 |
| uncharacterized cell_QUANTISEQ | -0.260 | p < 0.001 |
| T cell CD8+_MCPCOUNTER | 0.110 | p < 0.05 |
| NK cell_MCPCOUNTER | 0.156 | p < 0.01 |
| B cell_MCPCOUNTER | 0.255 | p < 0.001 |
| Monocyte_MCPCOUNTER | 0.265 | p < 0.001 |
| Macrophage/Monocyte_MCPCOUNTER | 0.265 | p < 0.001 |
| Myeloid dendritic cell_MCPCOUNTER | 0.346 | p < 0.001 |
| Endothelial cell_MCPCOUNTER | 0.330 | p < 0.001 |
| Cancer associated fibroblast_MCPCOUNTER | 0.363 | p < 0.001 |
| Myeloid dendritic cell activated_XCELL  B cell_XCELL | 0.227  0.137 | p < 0.001  p < 0.01 |
| T cell CD4+ naive_XCELL | 0.140 | p < 0.01 |
| T cell CD4+ central memory_XCELL | -0.145 | p < 0.01 |
| T cell CD8+_XCELL | 0.159 | p < 0.01 |
| T cell CD8+ central memory_XCELL | 0.113 | p < 0.05 |
| Common lymphoid progenitor_XCELL | -0.113 | p < 0.05 |
| Common myeloid progenitor_XCELL | 0.127 | p < 0.05 |
| Myeloid dendritic cell_XCELL | 0.182 | p < 0.001 |
| Endothelial cell_XCELL | 0.317 | p < 0.001 |
| Eosinophil_XCELL | 0.107 | p < 0.05 |
| Cancer associated fibroblast_XCELL | 0.372 | p < 0.001 |
| Granulocyte-monocyte progenitor_XCELL | 0.182 | p < 0.001 |
| Hematopoietic stem cell_XCELL | 0.408 | p < 0.001 |
| Macrophage_XCELL | 0.136 | p < 0.01 |
| Macrophage M1_XCELL | 0.141 | p < 0.01 |
| Macrophage M2_XCELL | 0.126 | p < 0.05 |
| B cell memory_XCELL | 0.119 | p < 0.05 |
| Monocyte_XCELL | 0.241 | p < 0.001 |
| T cell gamma delta_XCELL | -0.160 | p < 0.01 |
| T cell CD4+ Th1_XCELL | -0.318 | p < 0.001 |
| T cell CD4+ Th2_XCELL | -0.111 | p < 0.05 |
| immune score_XCELL | 0.213 | p < 0.001 |
| stroma score_XCELL | 0.381 | p < 0.001 |
| microenvironment score_XCELL | 0.352 | p < 0.001 |
| B cell_EPIC | 0.216 | p < 0.001 |
| Cancer associated fibroblast_EPIC | 0.268 | p < 0.001 |
| Endothelial cell_EPIC | 0.342 | p < 0.001 |
| Macrophage_EPIC | 0.205 | p < 0.001 |
| uncharacterized cell_EPIC | -0.372 | p < 0.001 |
|  |  |  |


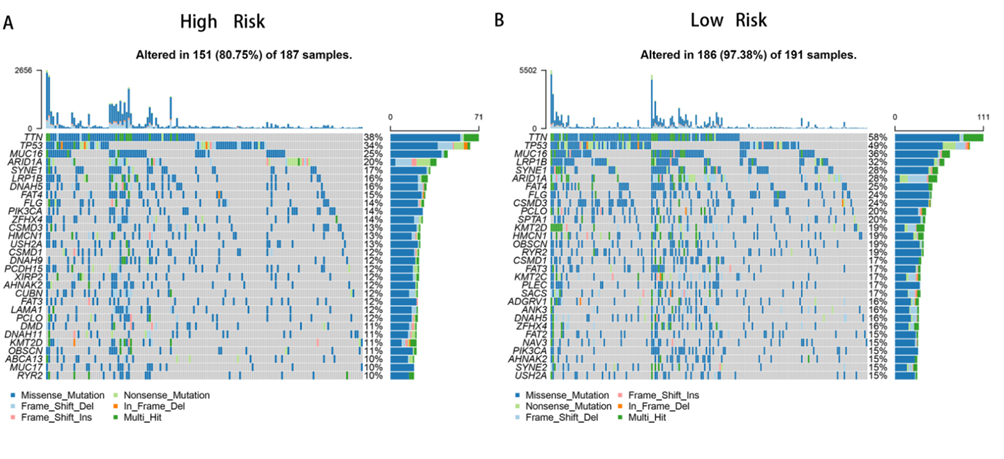


Supplementary Figure 1. Gene mutation landscapes of high-risk (A) and low-risk (B) groups.


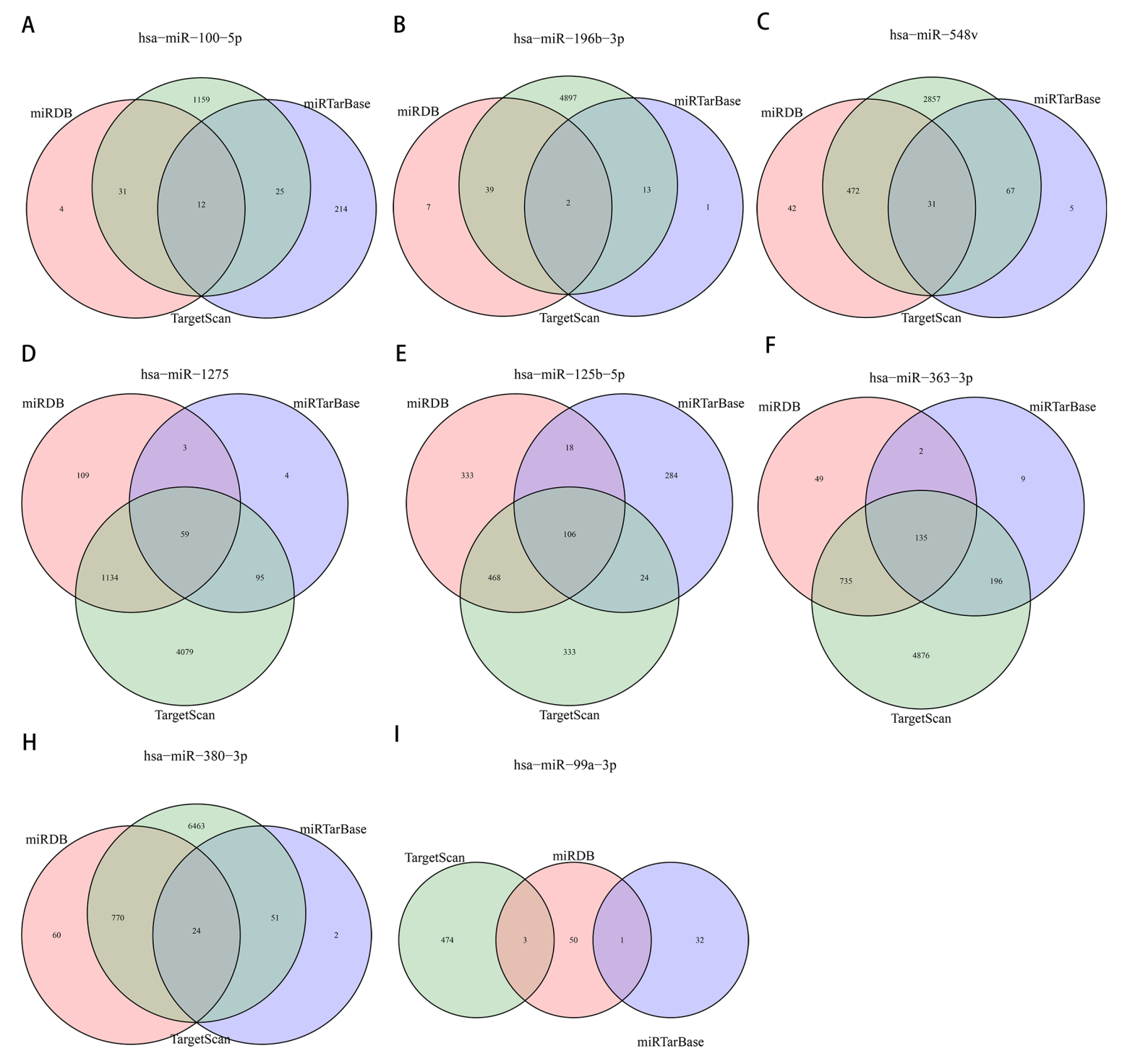


Supplementary Figure 2. Venn diagrams showing overlapping target genes according to TargetScan, MiRanda, and miRTarBase.
